# Pneumatosis intestinalis in oncologic patients: when should the radiologist not be afraid?

**DOI:** 10.1259/bjrcr.20160017

**Published:** 2016-07-27

**Authors:** Claudia Sassi, Milena Pasquali, Giancarlo Facchini, Alberto Bazzocchi, Giuseppe Battista

**Affiliations:** ^1^Department of Experimental, Diagnostic and Specialty Medicine (DIMES), Division of Radiology, S.Orsola-Malpighi Hospital, University of Bologna, Bologna, Italy; ^2^Diagnostic and Interventional Radiology, ‘Rizzoli’ Orthopaedic Institute, Bologna, Italy

## Abstract

Pneumatosis intestinalis (PI) is a term used to describe the presence of submucosal and subserosal gas in the gastrointestinal tract. It can occur as a primary disease or, more commonly, secondary to various other causes ranging from benign conditions to fulminant diseases. We present four cases of benign PI in patients being treated for various types of cancer. They had no abdominal symptoms, the physical examination was normal and PI was an isolated incidental CT finding in the absence of other signs of bowel wall distress. A conservative non-surgical approach was advocated and follow-up imaging documented the resolution of PI. The radiologist should recognize this condition in order to help the oncologist to interpret its clinical significance and avoid unnecessary surgical procedures.

## Background

Pneumatosis intestinalis (PI) is a term used to describe the presence of submucosal and subserosal gas in the wall of the gastrointestinal tract.^1^ PI is a radiological finding and not a diagnosis. It is usually diagnosed by plain abdominal radiography or CT scanning, but it could also be documented by MRI and ultrasonography.^2–4^

PI often has a dramatic radiological appearance and is most notably associated with life-threatening conditions such as bowel ischaemia, which often necessitates surgical intervention. It is generally considered to be a rare finding but can nonetheless be seen in a wide variety of conditions, some of which are benign.^1,5^ It can occur as a primary disease or, more commonly, secondary to various other causes ranging from benign conditions to fulminant diseases.^2^ Here we present four cases of PI in patients being treated for various types of cancer.

### Case report 1

**Figure 1.**
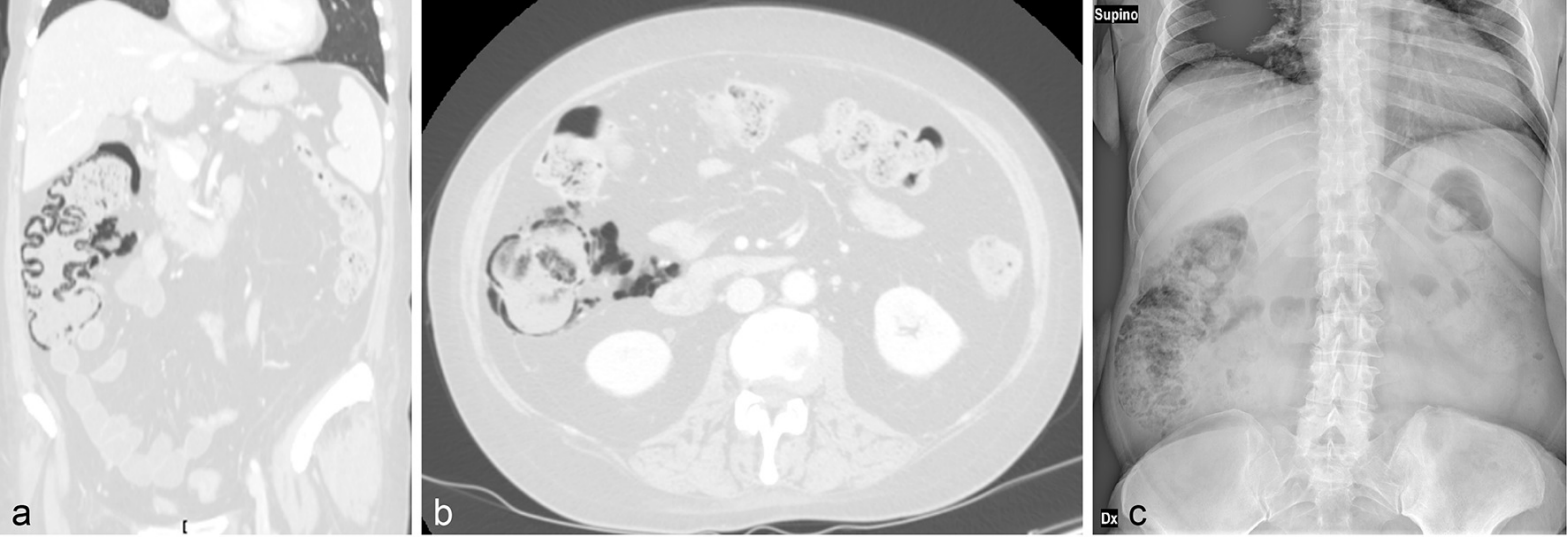
Case 1. A 56-year-old female with metastatic (liver, bones and brain) breast cancer receiving systemic chemotherapy plus corticosteroids. Restaging body CT scan, coronal (**a**) and axial (**b**) reformatted images; lung window. There is a moderate amount of pneumatosis in the mesentery and pericolic fat , and intramural gas along the caecum, ascending colon and a short section of the transverse colon. Bowel walls are not thickened or distended, haustra are regular and mesenteric vessels are patent. A plain abdominal film (c) taken 5 days later shows persistent asymptomatic pneumatosis.

A 56-year-old female with metastatic (liver, bones and brain) breast cancer treated with mastectomy and systemic chemotherapy underwent a restaging total-body CT scan. She was receiving a scheme of chemotherapy, including lapatinib, capecitabine, zoledronic acid and steroids (to reduce cerebral oedema). CT examination revealed a moderate amount of pneumatosis in the mesentery and pericolic fat, and intramural gas along the caecum and ascending colon (Figure 1), with the absence of other worrisome CT findings. The patient did not have abdominal pain or fever and had no history of any recent operative procedure or endoscopy. The oncologist, warned by the radiologist about the situation, decided to discontinue the steroids and follow a conservative approach, with clinical and radiological surveillance. The patient remained asymptomatic and follow-up imaging showed a slow resolution of PI.

### Case report 2

A 52-year-old female with breast cancer underwent left mastectomy and axillary dissection, and then received local radiotherapy. During the following 2 years, the cancer progressed, with metastases to the lymph nodes, bone and brain. For brain metastasis, she was treated with whole brain radiotherapy.

**Figure 2.**
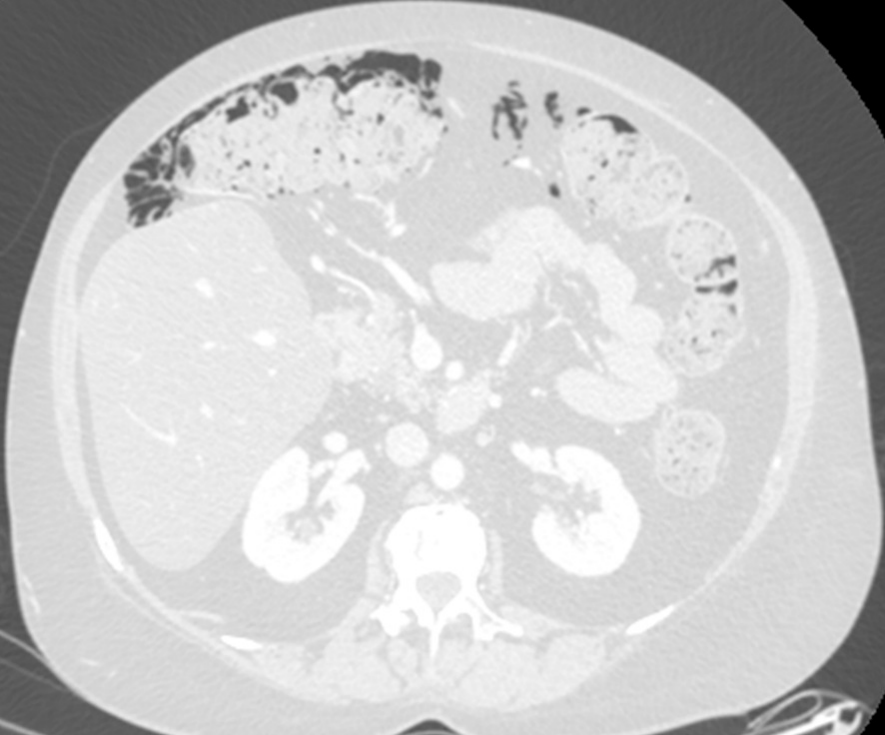
Case 2. A 52-year-old female with metastatic (lymph nodes, bone and brain) breast cancer, receiving chemotherapy and corticosteroids, submitted to body CT scan to rule out abdominal injury; axial CT image (detail of transverse colon), lung window. The image shows a moderate amount of intramural gas involving the caecum, ascending and transverse colon, with an extensive amount of gas in the mesenteric and pericolic fat; haustra are regular.

The patient was admitted to hospital to rule out injury following a minor trauma while she was being given systemic chemotherapy with docetaxel, trastuzumab and zoledronic acid in association with steroids to reduce cerebral oedema. A CT scan excluded any abdominal injury, but showed a moderate amount of PI along the caecum, and ascending and proximal transverse colon. On clinical examination, there were no signs of peritonism or sepsis, hence the surgical consultant recommended close conservative observation with supportive care alone. 5 days later, a follow-up CT scan of the abdomen showed an increase in the amount and extent of PI (Figure 2). Again, there were no other worrisome gastrointestinal findings on the CT and the patient was asymptomatic, thus steroids were discontinued and a conservative approach was followed. A CT scan performed a month later documented resolution of the PI.

### Case report 3

An 80-year-old male was found to have a pulmonary nodule that turned out to be an adenocarcinoma. The patient did not undergo surgery owing to the comorbidities he was suffering from, which included chronic obstructive pulmonary disease (COPD), diabetes and heart disease. He underwent stereotaxic radiotherapy with little benefit; indeed, the cancer progressed giving rise to lymph node and lung metastases bilaterally. He underwent several cycles of chemotherapy with vinorelbine, while receiving corticosteroids for COPD-related dyspnoea.

**Figure 3.**
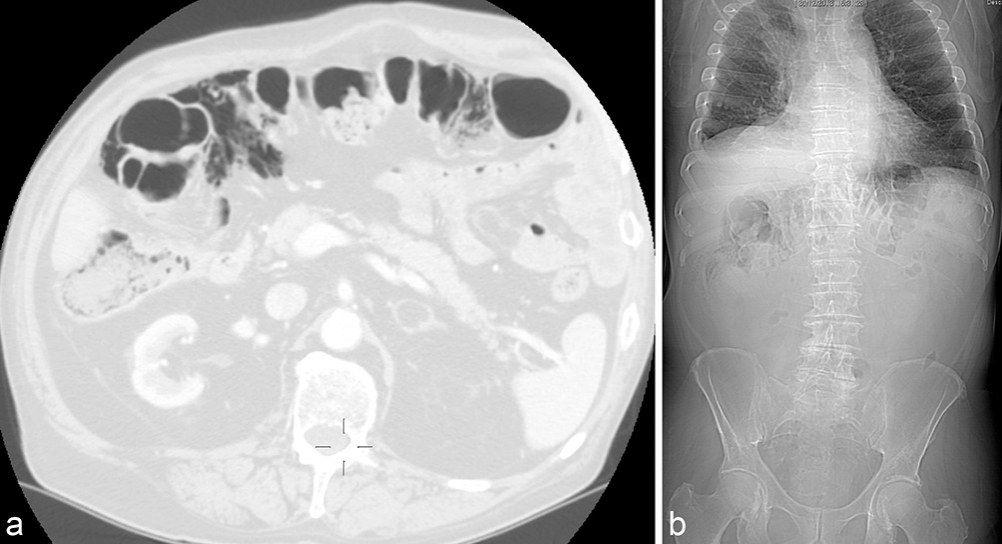
Case 3. An 80-year-old male receiving chemotherapy for lung adenocarcinoma and corticosteroids for chronic obstructive pulmonary disease-related dyspnoea. Chest CT; axial image, lung window. (**a**) Incidental finding of a large amount of gas in the adipose tissue along the caecum, and ascending and transverse colon; minimal intramural gas is confined to the caecum. The wall thickness is normal and haustra are regular. (b) Abdominal plain film performed a week later: persistence of a conspicuous amount of extravisceral gas along the caecum, right and transverse colon; no evidence of visceral distension.

A CT scan of the chest performed to evaluate worsening cough and dyspnoea showed a large amount of gas in the adipose tissue along the caecum, and the ascending and transverse colon (Figure 3a). The CT examination was extended to the abdomen and documented no other signs of bowel wall distress or perforation. The patient had no abdominal pain or fever; thus the surgical consultant suggested close observation alone. An abdominal plain film (Figure 3b) taken a week later documented the persistence of PI along the right and transverse colon. As the dyspnoea improved, the oncologist decided to gradually discontinue steroids and proceed with vinorelbine. A follow-up CT scan performed 4 months later showed improvement in the condition with a PI of minimum extent, limited to the caecum.

### Case report 4

**Figure 4.**
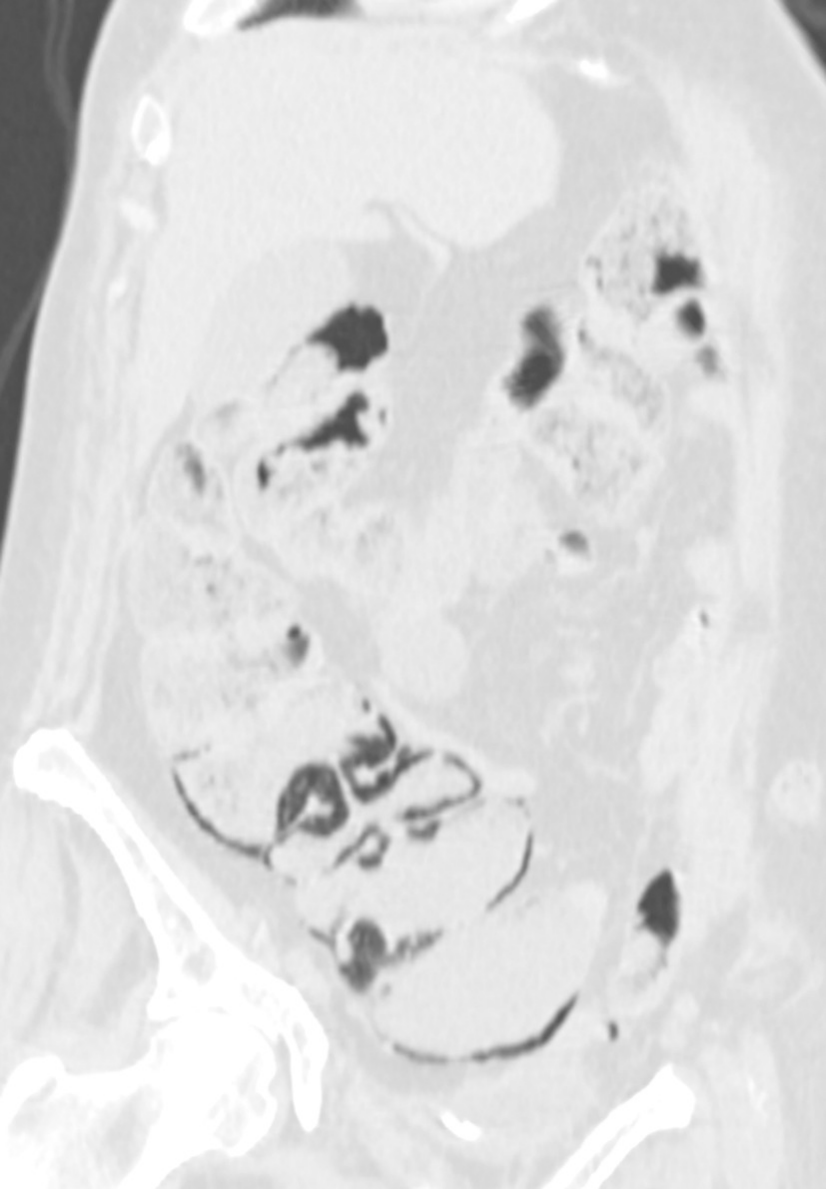
Case 4. A 57-year-old female receiving chemotherapy, including cetuximab after surgery, and radiotherapy for colorectal cancer. Restaging total-body CT scan; coronal oblique reformatted image; lung window. Moderate amount of intramural gas involving the caecum and ascending colon with the absence of pathological wall dilatation or thickening.

A 57-year-old female underwent anterior rectal resection and subsequent radiation therapy for colorectal cancer. A year later, owing to a locoregional recurrence of malignant disease, she underwent further surgery and radiotherapy, and was then started on systemic chemotherapy with 5-fluorouracil and oxaliplatin. Afterwards, she received capecitabine, then fluorouracil, folinic acid and irinotecan, and cetuximab. A subsequent restaging total-body CT scan showed moderate PI involving the caecum and the ascending colon (Figure 4) in the absence of other gastrointestinal worrisome findings. The patient had no signs of peritonism or sepsis. The surgical consultant recommended close observation with supportive care. The oncologist suspended cetuximab. A CT scan of the abdomen performed 3 days later showed a worsening of PI.The patient was still asymptomatic, thus a conservative approach was followed. Follow-up imaging documented a slow decrease of PI during the subsequent 2 months. The patient remained asymptomatic.

## Discussion

PI is a radiological finding and is caused by a wide variety of underlying gastrointestinal or extra-gastrointestinal diseases such as autoimmune (scleroderma, dermatomyositis), inflammatory (inflammatory bowel disease), infectious (*Clostridium difficile*, human immunodeficiency virus) or pulmonary (COPD) diseases; drugs (corticosteroids, immunosuppressive therapy); and trauma (blunt abdominal trauma, endoscopy).^6^

The aetiology and pathogenesis of PI are not fully understood but are likely multifactorial.^1–12^

PI probably occurs owing to a disruption of mucosal integrity, and there are two major theories about the source of the intramural gas:^13^

1. Bacterial theory: gas-producing bacteria translocate from the gastrointestinal lumen to the submucosal space through mucosal gaps or areas of enhanced permeability.
2. Mechanical theory: normal gas dissects from the lumen into the non-inflamed bowel wall, thanks to a loss of mucosal integrity.

CT features indicative of clinically worrisome PI are the presence of mesenteric stranding, bowel wall thickening and dilatation, ascites and confinement of the intramural gas to the small bowel. PI confined to the right colon is more frequently benign, whereas the presence of pneumoperitoneum is nearly always associated with every case of PI.^13^

We presented four cases of benign PI confined to the right colon (caecum, ascending colon and proximal transerse colon), incidentally observed as an isolated CT finding during routine examination in patients suffering from cancer without signs of peritonitis or sepsis, which resolved with supportive care alone and close observation.

The first two patients (case reports 1 and 2) received various chemotherapeutic agents plus corticosteroids to reduce cerebral oedema. Patients with cancer usually receive immunosuppressive or steroid therapies that may induce lymphoid depletion in Peyer’s patches, which impairs the gastrointestinal defence mechanism, reduces peristalsis and compromises the intestinal wall integrity.^7,8^ Some authors have observed an association between steroids and PI,^7,13^ with an improvement of PI after tapering steroids,^7^ but there is no statistical association between PI and the amount of steroids or chemotherapeutic agents (cisplatin, fluorouracil, gemcitabine, bevacizumab).^13^ Some authors have suggested that PI can be considered benign when confined to the colon and in the absence of “worrisome” CT scan findings such as bowel dilatation, bowel wall thickening, mesenteric stranding, ascites and portomesenteric venous gas.^13,14^ In our patients, PI met the criteria of benign PI and resolved after the discontinuation of steroids.

The third patient (case report 3) was receiving chemotherapy plus corticosteroids to reduce dyspnoea because he was suffering from COPD. PI may occur concomitantly with COPD, as well as with emphysema, chronic asthma and chronic bronchitis. The mechanical theory suggests that, in patients with COPD, an increase in intrapulmonary pressure from a ruptured alveolar or pulmonary bleb with coughing can either elevate intraluminal pressures, causing intramural dissection of air, or it can cause a pneumomediastinum with dissection of air retroperitoneally along vascular routes of the subserosal and submucosal areas of the bowel wall. A prolonged period ofbowel wall distension may cause abnormal intraluminal pressure, ischaemic mucosal changes and disordered peristalsis. A prolonged period of decreased peristalsis and elevated intraluminal pressure (induced by chemotherapy and corticosteroids) alone or in association with alveolar rupture could have allowed intramural dissection of gas in case report 3.^9^ Corticosteroids may have worsened this condition; in fact, PI resolved with improvement of dyspnoea and discontinuation of steroids.

The fact that in three of the four cases we presented, the discontinuation of steroids was accompanied by the remission of PI strengthens the association between PI and steroids.

In the fourth case (case report 4), the patient had previously undergone surgical resections and radiotherapy for colorectal cancer. Both these conditions may compromise the bowel wall microvasculature, predisposing to PI and ischaemia.^13^ In addition, the patient was receiving cetuximab, a chimeric immunoglobulin G1 monoclonal antibody that binds to epidermal growth factor receptors, thus inhibiting the ligand-induced phosphorylation of epidermal growth factor receptors, and possibly contributing to the gastrointestinal mucosal damage and alterations of permeability, leading to PI.^10,11^ In our patient (case report 4), the oncologist suspended cetuximab and PI gradually resolved.

The usage of biologically targeted agents in the treatment of cancer is likely to increase, especially in the molecular era of cancer and personalized medicine, so it is very important to pay attention to their possible adverse effects.^12,15,16^

In one case series, targeted molecular cancer chemotherapy with bevacizumab and sunitinib (24 patients) was associated with pneumatosis (10 patients) and bowel perforation (18 patients). The authors concluded that most patients can be treated conservatively after discontinuation of molecular targeted therapy. Continuing or restarting molecular targeted therapy can cause worsening, or recurrent pneumatosis or perforation.^17^

In our series, in two of four cases, we observed a worsening of the extent and amount of PI during the first week after diagnosis. PI remained an isolated CT finding, and the absence of signs of peritonism or sepsis was reassuring. A close clinical and radiological observation of benign PI is needed to identify cases that may turn out to be worrisome.

## Conclusions

Abdominal radiography and CT scanning are the most frequently used techniques for the diagnosis of PI. PI is usually thought to be worrisome, requiring surgical intervention, but it can be benign and treated conservatively.^13,15^

Identifying which patients can be managed conservatively relies on clinical, laboratory and mostly radiological findings.^13^ The radiologist should recognize this condition and rule out life-threatening causes such as intestinal ischaemia, bowel obstruction and perforation leading to intra-abdominal sepsis,^5^ thus assisting the treating oncologist in the interpretation of the clinical significance of PI.^15,16^

Some patients may have mild abdominal discomfort, which is usually related to the underlying associated medical condition. Physical examination is rarely abnormal.^14^

A conservative non-surgical approach is advocated in patients without signs of peritonitis or sepsis,^15^ but a close clinical and radiological observation is needed to recognize cases that may evolve into worrisome conditions.^16^ We observed an association between benign PI and the use of corticosteroids, but the small number of patients precludes any statistical significance.

In conclusion, a multidisciplinary interpretation of the clinical significance of PI is mandatory to avoid unnecessary surgical procedures.

## Learning points

1. Patients with cancer may present with PI that can be considered benign if it is an isolated CT finding and the patient is asymptomatic.
2. In patients with cancer, steroids and/or chemotherapeutic agents and/or surgical procedures possibly contribute to inducing gastrointestinal mucosal damage and alteration of permeability, leading to PI.
3. The radiologist should recognize CT findings of benign PI and report the same to the treating oncologist to avoid unnecessary surgical procedures.
4. Close observation of benign PI is advocated because it may worsen, proceeding to gastrointestinal necrosis/perforation.

## Consent

Written informed consent for the case to be published (including images, case history and data) was obtained from the patients for publication of this case report, including accompanying images.
